# Ultrasonography in assessing suspected bone fractures: a cross-sectional survey amongst German general practitioners

**DOI:** 10.1186/s12875-020-1078-5

**Published:** 2020-01-13

**Authors:** Gordian Lukas Schmid, Beatrice Kühnast, Marcus Heise, Tobias Deutsch, Thomas Frese

**Affiliations:** 1 Department of General Practice, Medical Faculty of the University of Leipzig, Philipp-Rosenthal-Str. 55, Leipzig, 04103 Deutschland; 2 Institute of General Practice and Family Medicine, Martin-Luther-University Halle-Wittenberg, Halle (Saale), Germany

**Keywords:** Bone fracture, Fracture diagnosis, Ultrasonography, Ultrasound imaging, Point of care, General practice

## Abstract

**Background:**

Over the last two decades, ultrasonography (US) has been shown to be an accurate tool for the diagnosis of suspected bone fractures; however, the integration of this application of US into routine care and outpatient settings needs to be explored. In this study, we surveyed German general practitioners (GPs) to assess their knowledge, attitudes, and utilization of US for the diagnosis of suspected fractures.

**Methods:**

Notification of the study, a self-designed questionnaire, and a reminder were mailed to 600 randomly selected GPs in Saxony and Saxony-Anhalt.

**Results:**

The response rate was 47.7% (*n* = 286), and respondents did not differ from the population of all GPs in respect to sex and practice type. Among GPs surveyed, 48.6% used an US device in their practice. On average, GPs diagnosed six patients with suspected fractures per month, yet only 39.3% knew about the possibility of ultrasonographic fracture diagnosis, and only 4.3% of GPs using US applied it for this purpose. Among participants, 71.9% believed that US is inferior to conventional X-rays for the diagnosis of bony injuries. Users of US were better informed of and more commonly used US for fracture diagnosis compared to non-users.

**Conclusion:**

The need to rule out possible fractures frequently arises in general practice, and US devices are broadly available. Further efforts are needed to improve the knowledge and attitudes of GPs regarding the accuracy of US for fracture diagnosis. Multicenter controlled trials could explore the safety, usefulness, and effectiveness of this still seldom used diagnostic approach for suspected fractures.

## Background

The use of ultrasonography (US) is increasing and progressively extending to new applications in almost all disciplines of modern medicine for procedural, screening, and diagnostic purposes [1]. As a diagnostic instrument, point-of-care US is substantially integrated into health care in hospital and outpatient settings [2].

In addition to the diagnostic evaluation of inner organs, blood vessels, muscles, and soft tissues, it has been demonstrated that US has a high sensitivity and specificity for the diagnosis of suspected bone fractures [3–7]. The detection of cortical discontinuities, step formation, and subperiosteal hematoma can be used as a diagnostic tool after acute trauma. In a systematic review Chartier et al. [7] reported on 30 publications showing a high sensitivity (64.7–100%) and specificity (79.2–100%) compared to plain radiographs in patients with suspected fracture of the long bones. These findings were confirmed recently by Champagne et al. [3]. Two additional systematic reviews [5, 6] focused on distal forearm fractures only and found an even higher accuracy. An overview for the diagnostic accuracy of ultrasonography used for different fracture locations and patient groups was published by Schmid et al. [4]. Accuracy was higher for fractures of the humerus, the forearm, the ankle, and the long bones in general, as well as fractures in children. Accuracy was lower for fractures of the short bones of the hands and feet, as well as fractures in adults.

The main advantages of US compared to conventional radiography are no radiation exposure, lower costs [5], and wider availability in non-hospital settings. Moreover, evidence suggests US may have higher accuracy than conventional radiographs for certain injuries, such as rib fractures [8] and early scaphoid fractures [9].

The use of US by general practitioners (GPs) across Europe varies widely, with fewer than 1% of GPs using US in Austria, Catalonia, Denmark, and Sweden, while 45% of GPs in Germany and 67% in Greenland use US [10]. Heidemann et al. [2] reported that ultrasound devices were available for about 70% of German GPs, although they found lower availability of US devices (32.1% of all GPs) for the area of former East Germany.

The vast majority of published studies on US-guided fracture diagnosis refer to observations in emergency departments [4], while very few studies report on outpatient settings [11, 12].

The aim of this exploratory study was to describe the utilization of US, knowledge and attitudes towards fracture diagnosis based on US, and referral behavior dealing with suspected fractures among German GPs. Differences between physicians using or not using US as a diagnostic tool and between GPs with long or short driving distances to the nearest radiographic unit were also investigated.

## Methods

### Sampling and design

We performed a cross-sectional survey of GPs from the federal states Saxony (*n* = 2733) and Saxony-Anhalt (*n* = 1445). Addresses were gained from publicly accessible registers, and the contacted GPs were selected from the register using random numbers (*n* = 400 Saxony, *n* = 200 Saxony-Anhalt). A response rate of about 25% was expected. Selected GPs first received a mailed notification of the study, which was followed by a mailed questionnaire 1 week later. A reminder was mailed 7 weeks after sending the questionnaire. Responses could be returned by fax or mail.

We received further pooled sociodemographic data of all GPs in Saxony and Saxony-Anhalt from the Associations of Statutory Health Insurance Physicians (Kassenärztliche Vereinigung), including average age, sex, and registration for reimbursement of US diagnostics.

### Questionnaire

The questionnaire was entirely self-designed by the research team, which included two social scientists, a medical student, and two experienced GPs who provided content-related input. A four-point scale was used for all questions regarding attitudes or personal assessments. To ensure face validity, the questionnaire was pre-tested among two groups of six GPs respectively, followed by additional feedback discussions. After minor revisions, the questionnaire was considered feasible by pre-testing physicians and could be completed in 5 min (Additional file 1: Questionnaire translated to English). The diagnostic scores and tools mentioned in the final question of the questionnaire were compiled through an unsystematic literature search.

### Statistical analyses

Data was analyzed using SPSS 25 (IBM SPSS Inc., Chicago, USA). Sociodemographic characteristics of the sample were presented descriptively (means, standard deviation, relative and absolute frequencies). Users and non-users of US were compared in bivariate cross tabulations regarding their knowledge and attitude towards US (relative and absolute frequencies). These differences were tested for statistical significance using chi-square tests (with Fisher-Exact correction as necessary). Further characteristics (e.g., suspected fractures after trauma, referring patients with suspected fractures) were analyzed using univariate statistics. The criterion of statistical significance has been set to an error probability of *p* < 0.05. Because of the small sample size, the inference statistics omit a correction for serial testing and are, therefore, exploratory.

### Ethics approval and consent to participate

According to the Model Professional Code for Physicians [13], an explicit ethics approval was deemed unnecessary for this study because no personal data of patients was collected. GPs were informed in writing about the use and publication of their anonymized data, and participants voluntarily consented by returning a completed questionnaire.

## Results

Of the 600 mailed questionnaires, 306 questionnaires were returned (51.0%), with 286 fully completed (47.7% response rate). Of the 286 completed questionnaires, 61 responses (21.3%) were received after a mailed reminder. A non-responder analysis showed no differences between responding GPs and non-responders with regard to sex, academic title, and specialization.

Sociodemographic data of the surveyed sample is given in Table 1. The study sample and the total population of GPs in Saxony and Saxony-Anhalt did not differ significantly in respect to sex and type of practice.

**Table 1:** Description of the study population: sex, type of practices, and approval for reimbursement of ultrasound diagnostic

	Sample (*n* = 286)	Total population (*n* = 4178)	95% C.I. within sample; test for differences between sample and total population
Sex			
Male	118 / 286 (41.3%)	1642 / 4178 (39.3%)	[35.6%; 47.0%] *p* = 0.489
Female	168 / 286 (58.7%)	2536 / 4178 (60.7%)	
Types of practices			
Single practice	202 / 286 (70.6%)	3126 / 4169 (75.0%)	[65.3%; 75.9%] *p* = 0.086
Medical care center	23 / 286 (8.0%)	343 / 4169 (8.2%)	[4.9%; 11.1%] *p* = 0.902
Joint practice	61 / 286 (21.3%)	700 / 4169 (16.8%)	[16.6%; 26.0%] *p* = 0.063
Approval of ultrasound	n.a.	1609 / 4178 (38.5%)	n.a.
Users of ultrasound	139 / 286 (48.6%)	n.a.	[42.8%; 54.4%]
Ultrasound unit in practices	159 / 286 (55.6%)	n.a.	[49.8%; 61.4%]

### Use of ultrasound

While only 38.5% of all GPs (*n* = 1609/4178) were registered for reimbursement of US diagnostics, 55.6% (*n* = 159/286) of surveyed GPs stated that there was a sonographic device available in their practices, and 48.6% (*n* = 139/286) used it. There were no differences in the availability of US devices between urban or rural areas (major cities: 55.8% (*n* = 43/77); small towns: 54.7% (*n* = 64/117); rural areas: 58.3% (*n* = 49/84); *p* = 0.876). The GPs using US reported performing on average 17 (mean = 16.7; median = 10; right-skewed distribution) examinations per week. The most frequently examined structures were the abdomen, the thyroid gland, and the kidneys (including the urinary passages). Only 4.3% (*n* = 6/138) of GPs using US stated that they regularly used US for the imaging of bone structures.

### Knowledge and beliefs

**Fig. 1:**
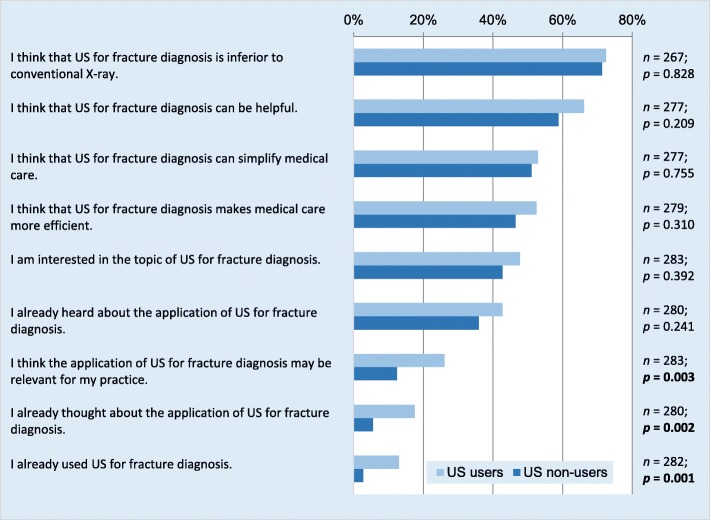
Knowledge, beliefs, and attitudes toward US for the diagnosis of bone injuries of GPs who use US compared with GPs who do not. Data is given as percentage of all responses to the respective question (*n*). The *p*-values refer to the corresponding null-hypotheses that there are no differences between both groups

The beliefs and attitudes regarding the use of US for suspected fractures among GPs are summarized in Fig. 1. The majority of respondents (71.9%, *n* = 192/267) believed that US is inferior to conventional X-rays for diagnosing bone injuries. While 39.3% (*n* = 110/280) of GPs stated to have knowledge of this imaging modality, only 19.1% (*n* = 54/283) judged it as relevant for their own practice, and 7.8% (*n* = 22/282) had any practical experience using US for fracture diagnosis.

GPs using US imaging in their practice significantly more often reported to have considered the application of US for fracture diagnosis, to have used US for this purpose, and to think that fracture diagnosis via US imaging might be relevant for their work.

### Diagnostic routines and referral behavior

Suspected fractures after trauma appear to be a frequent occurrence for consultation in general practice. Respondents estimated six patients per month visit their practice with a suspected fracture (mean = 6.4; SD = 10.2; median = 3; IQR = 9). We asked GPs about criteria taken into consideration when deciding whether to refer patients with suspected fractures for further imaging procedures. GPs considered anamnesis (98.9%, *n* = 282/285), persisting afflictions (97.9%, *n* = 279/285), dysfunctions (97.9%, *n* = 278/284), and pain (96.8%, *n* = 275/284) to be the most relevant criteria. The presence of swellings (93.0%, *n* = 264/284), their gut instincts (87.0%, *n* = 248/285) and hematoma (87.3%, *n* = 247/283) were used as decision criteria as well. Only 33.3% (*n* = 92/276) of GPs rated the use of scores or other clinical decision tools as “rather relevant” or “relevant”.

**Fig. 2:**
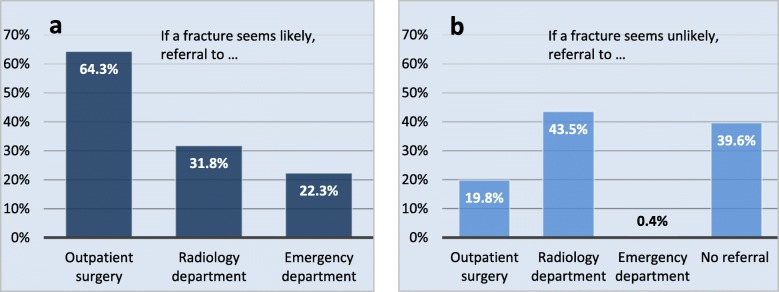
The referral behavior of GPs for patients with suspected bone fractures is visualized for the cases that a fracture was (**a**) likely or (**b**) unlikely. Data is given as percentage of all given answers to the respective question

The GPs were asked whether they would refer patients with suspected fractures and if so, to which specialty. The answers are summarized in Fig. 2. GPs from major cities were more likely to refer their patients to a radiologist than their colleagues from small towns or rural areas, whereas there was no statistically significant difference between referrals made to a surgeon or to the nearest emergency department (Table 2). No significant differences in referral behavior between users and non-users of US were found.

**Table 2:** Correlation between practice location and referral behavior of GPs treating patients with suspected fractures

	Major cities	Small towns	Rural areas	p-value
Number of practices (*n* = 278)	77 (27.7%)	117 (42.1%)	84 (30.2%)	
“If a bone fracture seems likely, where will you refer to?”				
Referral to				
- Outpatient surgery	60.5% *n* = 46/76	66.1% *n* = 76/115	65.5% *n* = 55/840	*p* = 0.711
- Radiologic department	51.3% *n* = 39/76	22.6% *n* = 26/115	26.2% *n* = 22/84	*p* < 0.001
- Emergency department	15.8% *n* = 12/76	27.0% *n* = 31/115	21.4% *n* = 18/84	*p* = 0.188

When asked to consider their level of confidence during the diagnostic process, 9.1% (*n* = 25/275) of the GPs indicated feeling totally uncertain, 24.4% (*n* = 67/275) rather uncertain, 49.8% (*n* = 137/275) rather confident, and 16.7% (*n* = 46/275) completely confident. When asked about shared decision making with patients, 98.6% (73.1%, *n* = 207/283 “yes, always”; 25.4%, *n* = 72/283 “yes, usually”) of the GPs indicated they discuss the likelihood of a bone fracture diagnosis with patients, and 92.1% (57.6%, *n* = 160/278 “yes, always”; 34.5%, *n* = 96/278 “yes, usually”) decided on further diagnostic procedures with patients or patients’ relatives.

### Influence of distance to radiological unit

GPs were asked if their practices were located in rural areas, small towns, or a major city. GPs were also asked to estimate the driving minutes to the nearest outpatient surgery, radiological department or emergency department. The answers are summarized in Table 3.

**Table 3:** Location of practices and estimated distances to radiologic units

	Major cities	Small towns	Rural areas
Number of practices (*n* = 278)	77 (27.7%)	117 (42.1%)	84 (30.2%)
Estimated driving times in minutes [median (min., max., IQR)]			
- Outpatient surgery	5 (0, 20, 7) *n* = 70	5 (0, 30, 5) *n* = 113	15 (0, 35, 10) *n* = 80
- Radiologic department	6 (0, 20, 5) *n* = 70	8 (1, 45, 10) *n* = 114	15 (0, 50, 10) *n* = 80
- Emergency department	10 (0, 30, 5) *n* = 77	8 (1, 40, 10) *n* = 117	15 (0, 100, 10) *n* = 84

GPs who stated that US for the diagnosis of suspected fracture was rather relevant or relevant for their own practice (“relevant” *n* = 54 vs. “not relevant” *n* = 229) were, on average, situated farther from their nearest surgeon (mean [SD]: 13.0 [±9.0] vs. 8.8 [±6.6] minutes; *F* = 4.89, *p* = 0.003), radiological (mean: 14.7 min [SD = 9.9] vs. 10.9 [±8.5] minutes; *F* = 6.13, *p* > 0.001) or emergency department (16.4 [±14.9] vs. 11.1 [±7.7] minutes; *F* = 3.17, *p* = 0.025). A similar correlation was found for GPs who had considered using US for fracture diagnosis. There were no correlations between the distances to the next surgeon, radiology, or emergency department and the use of US or referral behavior.

## Discussion

In this study, we surveyed a random sample of GPs in Germany (Saxony and Saxony-Anhalt) regarding their knowledge, attitudes, and utilization of US for the diagnosis of suspected bone fractures. Although half of the responding doctors used US as diagnostic imaging modality in their daily routine, its accuracy and potential for detecting bone injuries were grossly underestimated. Around one-third of those surveyed knew that US can be used in fracture diagnosis. Yet, more than two-thirds of GPs believed US was inferior to conventional radiographs, and fewer than 8% had experience with ultrasonographic investigation of bone structures.

In a search of recent literature, we found no comparable studies examining the use of US for fracture diagnosis in general practice. Jacobs et al. [14] investigated the effect of the introduction of teleradiology on the number of performed radiographic examinations for suspected fractures in a remote general practice. The possibility of making a diagnosis by the GP reduced the number of unnecessary referrals to the hospital, and more patients with fractures were treated in the general practice rather than the hospital. A similar effect might be presumed for integrating US as an addition to the physical examination of suspected bone fractures.

### Implications for future practice

We found that GPs regularly encounter suspected bone fractures, and half of all GPs own an US device. Further evaluation of US as a diagnostic tool for suspected fractures in general practice seems promising and possible.

For safe and cost-effective patient care, an accurate imaging modality to rule out suspected fractures is needed. Today, conventional radiographs are most frequently utilized to make a diagnosis [7]. Radiographs are widely available in Germany, as illustrated by our finding that 75% of the GPs from rural areas reported the closest radiology department to be reachable in less than 20 min driving by car.

Our findings show that 43% of GPs refer their patients to the radiology, even if a fracture seems unlikely. This finding suggests a demand for specific tools with a high negative predictive value to help rule out fractures with sufficient certainty. Consequently, the high number of radiographs might be reduced.

There are clinical scores and suggestions for evidence-based diagnostic algorithms for certain fracture sites (e.g., Ottawa Foot and Ankle Rules [OFAR]) which help to enhance the pretest probability for radiographic imaging. These tools might help to reduce the radiation exposure for patients, especially for children and pregnant women who are particularly vulnerable. However, this survey revealed that GPs use such scores only rarely to support their suspected diagnoses. The potential of combining clinical scores and US was pointed out by Jonckheer et al. [15] and investigated by Canagasabey et al. [16] and Tollefson et al. [17], who combined the sensitive but unspecific OFAR with bedside sonography, which thereby increased the specificity of the testing. It would be helpful to formulate such clear diagnostic tools to include US for general practice and other outpatient settings and reduce the number of radiographic examinations, which would avoid unnecessary costs and radiation exposure. A focused training for physicians would further facilitate greater use of US based fracture diagnosis. Several pilot studies demonstrated, that the skills necessary to conduct a structured examination can be taught within a short time [18–20].

### Implications for future research

Potential benefits of utilizing US for the diagnosis of suspected fractures in general practice need to be carefully investigated. It remains to be seen whether fracture US in general practice can improve patients’ safety and comfort in addition to reducing costs. The diagnostic process for suspected fractures with US should therefore be assessed in prospective multicenter studies. Further studies of test performance in general practice or other outpatient settings with a relatively low pre-test probability for fractures should also take into account organizational and financial aspects, safety, and practicability.

Two-thirds of GPs believed US to be helpful for fracture diagnosis, indicating openness for this new application of the well-known diagnostic device. However, 71.9% of GP respondents had greater trust in conventional radiographs than in sonographic imaging to confirm suspected fractures. In addition, GPs indicated radiological departments were easily accessible, with a mean driving time of only 11 min from their general practice. This finding indicates the acceptance of GPs for US as diagnostic tool for suspected fractures may only be improved by providing clear evidence and convenient recommendations for daily practice.

### Strengths and limitations

To our knowledge, this study is the first to investigate the attitudes and beliefs of GPs toward US for fracture diagnosis.

However, this study has several limitations. The questionnaire was self-designed, and questions assessing the feelings and attitudes of GPs toward fracture diagnosis and the use of US for this purpose were not validated. This might limit the reliability of those results. Because of the cross-sectional design, we relied on estimates reported by GPs of the incidence of suspected fractures.

In addition to the actual use of US in the daily practice, it would have been interesting to assess how many GPs underwent an explicit training for US based fracture diagnosis.

The response rate achieved in our study was relatively high compared to similar recently published surveys among German GPs [21–24] and acceptable considering international findings [25]. Nonetheless, it was lower than 50%, and bias due to an overrepresentation of those GPs interested in the topic cannot be excluded.

Our sample may not be representative of all German GPs, as regional disparities in the presence of US devices in general practices across the federal states have been described [2]. However, it is possible that physicians who are routinely using US in their practice are overrepresented among respondents (US device available for 55.6% of the responding GPs vs. 32.1% as reported by Heidemann et al. [2]). These physicians may overestimate the potential and benefits of US for fracture diagnosis. Yet, given these findings of low utilization and poor knowledge of US for suspected fractures, it is reasonable to assume the need for information and clear evidence in this field is even higher for the total GP population in Germany.

## Conclusion

Ruling out suspected bone fractures is a frequent task in general practice, and US devices are available for half of the GPs in Germany. Further efforts are needed to improve the knowledge and attitudes of GPs regarding the accuracy of US for fracture diagnosis. Multicenter controlled trials could explore the safety, usefulness, and effectiveness of this still seldom used diagnostic approach for suspected fractures.

## Supplementary information

**Additional file 1.** Questionnaire translated to English.

## Data Availability

Anonymized survey responses obtained through this study are available from the corresponding author on reasonable request.

## Abbreviations

GP: General practitioner
US: Ultrasonography
