# Supplementary material for: Ultrasonography in assessing suspected bone fractures: a cross-sectional survey amongst German general practitioners
Source: BMC Fam Pract. 2020 Jan 13;21:9. doi: 10.1186/s12875-020-1078-5 (PMC6958739; doi:10.1186/s12875-020-1078-5)
Supplement: Supplementary file 1 — Additional file 1. Questionnaire translated to English. [file 12875_2020_1078_MOESM1_ESM.pdf]

Application of medical ultrasound imaging for bone fracture diagnostics in general practice

Attention: We kindly ask you to answer all questions in this form completely. Thank you!

Personal details:

Age: ○ < 30 years ○ 30 – 40 years ○ 40 – 50 years ○ 50 – 60 years ○ > 60 years
Sex: ○ female ○ male

In which university did you finish your medicine studies? ________________________________

Highest academic degree: ○ state examination ○ diploma ○ doctorate ○ habilitation

Acquired specializations (please name all and the year of acquisition):

Specialist for ______________________________ , since: ________ (year)

Specialist for ______________________________ , since: ________ (year)

Acquired special designations (please name all and the year of acquisition):

Designation for ______________________________ , since: ________ (year)

Designation for ______________________________ , since: ________ (year)

Designation for ______________________________ , since: ________ (year)

Memberships: ○ DEGAM ○ SGAM ○ Hausärzteverband ○ none

When did acquire your latest certificate of advanced education? ___ (year) ○ I never acquired one.

Practice type: ○ employed since: ________ ○ licensed since: ________

Office details:

Office type: ○ single practice ○ joint practice ○ medical care unit

How would you describe the area where most of your patients come from?
○ major city ○ small-town ○ rural area

Distance to the next hospital (driving minutes by car from office): __________________ min.
Distance to the next radiologist (driving minutes by car from office): __________________ min.
Distance to the next surgeon (driving minutes by car from office): __________________ min.

Application of medical ultrasound imaging in the office:

Do you own a sonographic device? ○ yes ○ no

In case you own a device, who is the main user? ○ myself ○ others

In case you own a device, how many examinations are performed in an average week?
About ________________ examinations

In case you own a device, which organs or structures are most frequently examined?
(multiple selection is possible):

○ thyreoid gland ○ upper abdomen ○ vessels
○ kidneys / urinary tracts ○ heart (TTE) ○ joints
○ bone structures ○ others: ______________________________

Epidemiology:

How many patients with potential bone fractures after a trauma do you examine?
______ x / month if less than 1 x / month: ___________ / year

While diagnosing potential bone fractures, I feel:
○ insecure ○ rather insecure ○ rather confident ○ confident

I consider the diagnostic process to rule out potential bone fractures as:
○ very inconvenient ○ rather inconvenient ○ rather convenient ○ convenient

Please turn over!

Topic assessment:

I am interested in the topic of ultrasonographic imaging for the diagnosis of potential bone injuries.
○ not true ○ rather not true ○ rather true ○ true

I think that the application of ultrasonographic imaging for the diagnosis of potential bone injuries may be relevant for my practice.
○ not true ○ rather not true ○ rather true ○ true

I think that ultrasonographic imaging for the diagnosis of potential bone injuries can be helpful.
○ not true ○ rather not true ○ rather true ○ true

I think that ultrasonographic imaging for the diagnosis of potential bone injuries can simplify medical care.
○ not true ○ rather not true ○ rather true ○ true

I think that ultrasonographic imaging for the diagnosis of potential bone injuries makes medical care more efficient.
○ not true ○ rather not true ○ rather true ○ true

I think that ultrasonographic imaging for the diagnosis of potential bone injuries is inferior to conventional X-ray.
○ not true ○ rather not true ○ rather true ○ true

I already heard about the application of ultrasonographic imaging for the diagnosis of potential bone injuries.
○ not true ○ rather not true ○ rather true ○ true

I already thought about the application of ultrasonographic imaging for the diagnosis of potential bone injuries.
○ not true ○ rather not true ○ rather true ○ true

I already used ultrasonographic imaging for the diagnosis of potential bone injuries.
○ not true ○ rather not true ○ rather true ○ true

Referral criteria:

If a bone fracture seems **likely**, where will you refer to?
○ radiologist ○ general surgeon ○ emergency department

If a bone fracture seems rather **unlikely**, where will you refer to?
○ radiologist ○ general surgeon ○ emergency department ○ no referral

If a bone fracture seems rather **unlikely**, how secure do you feel about the decision against further diagnostics:
○ insecure ○ rather insecure ○ rather confident ○ confident

Do you discuss the likelihood of a bone fracture together with the patient (and his/her relatives)?
○ no, never ○ no, usually ○ yes, usually ○ yes, always

Do you decide about further diagnostics together with the patient (and his/her relatives)?
○ no, never ○ no, usually ○ yes, usually ○ yes, always

How important are the following criteria for the decision making process of a referral concerning posttraumatic imaging to you?

anamnesis	○ irrelevant	○ rather irrelevant	○ rather important	○ important
pain	○ irrelevant	○ rather irrelevant	○ rather important	○ important
swelling	○ irrelevant	○ rather irrelevant	○ rather important	○ important
haematoma	○ irrelevant	○ rather irrelevant	○ rather important	○ important
functio laesa	○ irrelevant	○ rather irrelevant	○ rather important	○ important
persistant afflictions	○ irrelevant	○ rather irrelevant	○ rather important	○ important
own instincts	○ irrelevant	○ rather irrelevant	○ rather important	○ important
scores / clinical decision tools	○ irrelevant	○ rather irrelevant	○ rather important	○ important

Which of the following clinical scores for the assessment of potential bone fractures do you know?
(multiple selection is possible)

○ Neer Score ○ Score by Weseley et al. ○ Score by Solgaard ○ other
○ Ottawa Ankle Rule ○ Ottawa Foot Rule ○ Canadian Cervical Spine Rules ○ none
○ Ottawa Knee Rule ○ Pittsburgh Knee Rule ○ Harris Hip Score

Thanks for your support!
